# Identifying Therapeutic Targets for Spinocerebellar Ataxia Type 3/Machado–Joseph Disease through Integration of Pathological Biomarkers and Therapeutic Strategies

**DOI:** 10.3390/ijms21093063

**Published:** 2020-04-26

**Authors:** Yu-Shuan Chen, Zhen-Xiang Hong, Shinn-Zong Lin, Horng-Jyh Harn

**Affiliations:** 1 Bioinnovation Center, Buddhist Tzu Chi Medical Foundation, Hualien 97002, Taiwan; 2 Department of Medical Research, Hualien Tzu Chi Hospital, Buddhist Tzu Chi Medical Foundation, Hualien 97002, Taiwan; 3 Department of Neurosurgery, Hualien Tzu Chi Hospital, Buddhist Tzu Chi Medical Foundation, Hualien 97002, Taiwan; 4 Department of Pathology, Hualien Tzu Chi Hospital, Tzu Chi University, Buddhist Tzu Chi Medical Foundation, Hualien 97002, Taiwan

**Keywords:** spinocerebellar ataxia type 3/Machado–Joseph disease, therapeutic strategies, pathological biomarkers

## Abstract

Spinocerebellar ataxia type 3/Machado–Joseph disease (SCA3/MJD) is a progressive motor disease with no broadly effective treatment. However, most current therapies are based on symptoms rather than the underlying disease mechanisms. In this review, we describe potential therapeutic strategies based on known pathological biomarkers and related pathogenic processes. The three major conclusions from the current studies are summarized as follows: (i) for the drugs currently being tested in clinical trials; a weak connection was observed between drugs and SCA3/MJD biomarkers. The only two exceptions are the drugs suppressing glutamate-induced calcium influx and chemical chaperon. (ii) For most of the drugs that have been tested in animal studies, there is a direct association with pathological biomarkers. We further found that many drugs are associated with inducing autophagy, which is supported by the evidence of deficient autophagy biomarkers in SCA3/MJD, and that there may be more promising therapeutics. (iii) Some reported biomarkers lack relatively targeted drugs. Low glucose utilization, altered amino acid metabolism, and deficient insulin signaling are all implicated in SCA3/MJD, but there have been few studies on treatment strategies targeting these abnormalities. Therapeutic strategies targeting multiple pathological SCA3/MJD biomarkers may effectively block disease progression and preserve neurological function.

## 1. Introduction

Spinocerebellar ataxia type 3 (SCA3), also named Machado–Joseph disease (MJD), is an autosomal dominant cerebellar ataxia associated with the expansion of the ATXN3 exon 10 CAG repeat to 45 copies or more, resulting in a mutant ataxin-3 protein with an expanded poly-glutamine (polyQ) tract. Type 3 is the most common SCA worldwide, with an estimated prevalence of 1–5/100,000. Mainland China has the highest prevalence of SCA3 among SCAs, accounting for 62.6% of all cases, followed by Brazil (59.6% of all SCA cases), Japan (43%), and Germany (42%) [1]. Type 3 is also the most prevalent SCA in Taiwan, accounting for 47.3% of all cases [2]. Age at onset is negatively correlated with polyQ length, directly implicating the CAG repeat expansion in disease pathogenesis [3]. Gait ataxia is the most common symptom of SCA3, and average survival duration is only 21.18 years after symptom onset [4].

Although many clinical trials have been conducted on various candidate SCA3 treatments, there is still no United States Food and Drug Administration (FDA)-approved drug for this disease. The majority of current therapeutic strategies are aimed at specific symptoms, such as supportive treatment and physical therapy for motor dysfunction. Preclinical studies on potential therapeutic strategies for SCA3/MJD were recently reviewed in detail by Matos and colleagues [5]. As there are currently no therapies in practice or current development that appear likely to revolutionize treatment in the short term, it may be helpful to pursue alternative strategies, such as more intensive biological marker screening of presymptomatic and symptomatic patients to identify novel target molecules and pathogenic pathways. However, few reviews have focused on the relevance of biological markers to potential therapeutic mechanisms. Therefore, we have examined the associations between biological markers and therapeutic strategies to provide additional clues to novel SCA3/MJD treatments. Moreover, completed, ongoing, and suspended clinical trials are included in this discussion to highlight less studied, controversial, and otherwise neglected therapeutic strategies.

## 2. Content

To fully explore potential therapeutic mechanisms for SCA3, clinical trials are summarized in the first section and associations between biomarkers and pre-clinical strategies in the second section.

### 2.1. Update on Clinical Trials for SCA3/MJD Treatment

As of January 2020, 110 clinical trials of SCA treatments have been completed or are ongoing. Among them, 23 are drug trials specific for SCA3 registered at ClinicalTrials.gov (summarized in Table 1). Synthetic chemical drugs and stem cells are the two major treatment types tested. Tested drugs include neurotransmitter modulators, ion transport inhibitors, growth factors, histone deacetylase (HDAC) inhibitors, and autophagy enhancers, while adipocyte-derived and umbilical cord-derived stem cells are two major sources for cell-based treatment of SCA3.

#### 2.1.1. Neurotransmitter Modulators

Most therapeutics employed for SCA3 modulate neurotransmitter signaling, including 5-HT1A receptor agonists, nicotinic acetylcholine receptor agonists [10], ion transport inhibitors [12], potassium channel activators, glutamatergic transmission inhibitors, and various other neurotransmitter receptor agonists or antagonists. Unfortunately, neither the ion transport inhibitor lithium carbonate nor the potassium channel blocker dalfampridine has demonstrated therapeutic efficacy against disease progression compared to placebo.

Buspirone and tandospirone are 5-HT1A receptor agonists currently marketed as anti-anxiety and antidepressant medications. Modulation of serotonin signaling is the major therapeutic strategy against insomnia and depression. In the cerebellum, serotonin signals to Purkinje cells (PCs) via 5-HT1A receptors. In addition, buspirone activates dopamine receptors and inhibits glutamate release at cerebellar parallel fiber–PC synapses. Buspirone has been shown to improve gait ataxia, leg pain, anorexia, and insomnia in cerebellar ataxia and SCA3/MJD patients [6,7,8].

The α4β2 neuronal nicotinic acetylcholine receptor agonist varenicline, in current clinical use as a smoking cessation aid, has also been tested in a Phase 2 trial. The trial included 20 SCA3 patients (mean age = 51 ± 10.98 years; mean disease duration = 14 ± 9.82 years; mean scale for assessment rating of ataxia [SARA] score = 16.13 ± 4.67), of which data from 18 patients were analyzed in period I. Varenicline significantly improved axial symptoms and rapid alternating movements [10], while the most common side effect was nausea. In another study, however, only one patient out of seven completed the trial due to intolerable nausea, insomnia, and/or depression. Nonetheless, the treated patient did show slightly improved axial symptoms [15]. The muscarinic antagonist metixene HCl has also been examined in an animal model of SCA3, but it increased sodium dodecyl sulfate-insoluble aggregation of ataxin-3 compared to no treatment [16].

Numerous clinical trials have investigated the therapeutic efficacy of drugs that disrupt glutamatergic neurotransmission as overstimulation and the ensuing glutamate-mediated excitotoxicity are implicated in many neurological disorders. A trial in 2010 (NCT01104649) reported that the glutamate release inhibitor riluzole reduced the SARA score in one-half of patients tested, without severe side effects [13]. In 2018, Phase 2 trials were initiated for two riluzole derivatives, troriluzole (a tripeptide derivative) and BHV-4157 (a pro-drug of riluzole).

Thyrotropin-releasing hormone (TRH) is a pleiotropic modulator of downstream hormones and neurotransmitters, as well as a putative neurotransmitter. It is also among the first drugs approved in Japan for spinocerebellar degeneration. In the cerebellum, TRH promotes long-term depression of excitatory glutamatergic transmission from parallel fibers to PCs by reducing α-amino-3-hydroxy-5-methyl-4-isoxazolepropionic acid (AMPA)-type receptor activity via the NO/cGMP signaling pathway [17]. Thus, TRH may enhance motor learning, which is dependent on long-term depression at parallel fiber–PC synapses, and suppress glutamatergic excitotoxicity of PCs. A Phase 3 trial in 2013 (NCT01970098) found that the TRH derivative rovatirelin (KPS-0373) improved severe ataxia in SCA31 and SCA6 patients and that these effects were more prominent than in patients with less severe ataxia [18]. However, the initial trials on TRH were initiated over 30 years ago, and so the efficacy of TRH derivatives warrants re-examination [19]. The TRH analog C-Trelin orally disintegrated (OD) tab has been proposed for the treatment of spinocerebellar degeneration, including SCA3, and a Phase 4 trial was registered in 2019 (NCT04107740).

#### 2.1.2. Growth Factors

Insulin-like growth factor-1 (IGF-1) has been tested for the treatment of SCA3 and SCA7. While SARA scores were reduced in the treatment group, the study was open-labeled and uncontrolled, and thus could not exclude a placebo effect [9]. Aside from that study, IGF-1 treatment has only been examined for growth failure in ataxia telangiectasia patients (NCT01052623).

Nerve growth factor has been tested in SCA3 patients who were administered an intramuscular injection. The treatment period was only 28 days in an open-label study. Decreased SARA and improved stance, speech, finger chase, fast alternating hand movements, and heel–shin slide (*p* = 0.001) were observed [20].

#### 2.1.3. HDAC Inhibitors

Valproic acid (VPA) is a pan-HDAC inhibitor used to treat bipolar disorder and epilepsy. Administration to SCA3 patients resulted in a larger decrease in the SARA score (−2.05) than in response to placebo (−0.75). Valproic acid also has been applied for the treatment of other neurodegenerative diseases such as amyotrophic lateral sclerosis (ALS), stroke, and Alzheimer’s disease (AD) and has demonstrated neuroprotective, anti-inflammatory, and angiogenic efficacy [11]. Sodium phenylbutyrate is another potent HDAC inhibitor, but it has not met regulatory requirements for human study.

#### 2.1.4. Autophagy Enhancers

The chemical chaperone trehalose (Cabaletta) acts as an mTOR pathway-independent autophagy inducer. A trial sponsored by Bioblast Pharma Ltd. found that trehalose (13.5 or 27 g/week) stabilized the SARA scale without adverse effects. The Bcr-Abl tyrosine kinase inhibitor (TKI) nilotinib was also shown to induce autophagy through the AMP-activated protein kinase (AMPK) pathway. A Phase 2 trial has been registered (Bcr-Abl TKI, NCT03932669) but was not recruiting as of 2019.

#### 2.1.5. Stem Cells

An open-label study in 2012 from Taiwan applied allogeneic adult adipose-derived mesenchymal stem cells (MSCs) for treatment of SCA3 as such cells have been shown to protect neurons through trophic factor production and by reducing reactive oxygen species (ROS) generation. Furthermore, the use of an allogeneic cell source rather than autologous cells obviates the possibility of poor efficacy due to the underlying genetic disorder. Transplantation increased both brain glucose metabolism and neurotrophic factor production without adverse events (NCT01649687) [14]. Two Phase 2 trials using human adipose tissue stem cells (NCT02540655) or umbilical cord MSCs, NCT03378414) are still ongoing for SCA but have not recruited patients since 2015 and 2017, respectively.

Riluzole and the TRH analog C-Trelin OD appear to be the most successful candidate SCA treatments, as evidenced by recent Phase 3 and Phase 4 clinical trial registrations. However, the potential of the other candidate drugs is unclear in many cases. Furthermore, the choice of test drug was not always based on a known pathogenic mechanism or biomarker for SCA3. Alternatively, studies on biological markers for SCA3 may yield more promising candidates for preclinical studies and clinical trials. The following sections provide a comprehensive discussion of new therapeutic strategies to treat SCA3 based on insights from biomarker identification.

### 2.2. Experimental Therapeutic Strategies for SCA3/MJD

Matos and colleagues [5] have reviewed current therapeutic approaches, although not all have been tested in clinical trials. Therefore, we assume some important issues have gone unreported. Biomarkers upregulated or downregulated in SCA3 animal models are summarized in Table 2 together with those detected in SCA3 patient specimens. The following sections will focus on novel treatment strategies based on biomarker expression, including RNAi-mediated knockdown of ataxin-3, reducing cleaved protein formation and aggregation, anti-inflammation, mitigating oxidative stress, and rescue of cellular dysfunction.

#### 2.2.1. RNAi Silencing of Ataxin-3 Expression

Strategies for ataxin-3 silencing include transfection of allele-specific and -nonspecific RNAi constructs targeting the 3′UTR of ATXN3 mRNA. Specific knockdown of mutant ATXN3 could be performed by targeting a single nucleotide polymorphism allele or using exon-skipping constructs targeting exon 9 or 10 [54,55]. Alternatively, allele-nonspecific silencing of mutant and wild-type ATXN3 would also achieve the goal of reducing protein aggregation and ensuing neuropathology as wild-type ATXN3 knockdown would not aggravate SCA3 pathology [56].

Shi and colleagues found lower expression levels of the micro RNAs (miRNA) miR-29a, miR-25, and miR-125b in patient sera compared to controls [21]. However, only transfection of a miR-25 mimic reduced expression of wild-type ataxin-3 or mutant ataxin-3 in HEK293T, SH-SY5Y, and SCA3/MJD model cells [22]. In addition to miR-25, species miR-9, miR-181a, and miR-494 have been demonstrated to bind the 3′-UTR of ATXN3 mRNA and inhibit ataxin-3 expression [23]. Downregulation of miR-9 and miR-181a have been confirmed in cerebrospinal fluid-derived exosomal miRNA arrays from human MJD patients and animal models [23,24].

These RNAi constructs could be delivered to patients using vectors such as adeno-associated virus (AAV) [57] and lentivirus [58] or by non-viral systems such as lipid nanoparticles [59]. In either case, efficient long-term expression of the RNAi is required to suppress protein aggregation. Nóbreg and colleagues reported long-term suppression (20 weeks post-injection) using a lentiviral vector encoding short hairpin RNA (LV-shmutatx3) [58]. Although miRNA-based therapeutics have not yet been applied for SCA3/MJD treatment, they have been examined in clinical trials for other diseases such as inherited Alport’s syndrome and ALS. For example, the miR-29b mimic MRG-201 has been shown to restore miR-29b activity for antifibrotic activity in the treatment of fibrosis [60]. Based on these findings, the application of miRNA-based therapeutics for SCA3/MJD appears feasible.

#### 2.2.2. Reduced Cleavage Protein Formation

Protein aggregation in the cytosol or nucleus is frequently the result of aberrant proteolytic cleavage. Ataxin-3 is cleaved by endogenous enzymes such as calpain and caspase to form N-terminal and C-terminal ataxin-3, both of which have polyQ regions [61]. The C-terminal ataxin-3 also contains a nuclear localization signal and so may form nuclear inclusions that disrupt DNA repair and transcriptional regulation [61]. In fact, a smaller fragment (36 kDa) missing the N-terminal was enriched in the nuclear fraction of patient samples [25,62].

The calpain inhibitor calpastatin was found to reduce aggregated ataxin-3 inclusions in three MJD patients (by 67%, 25%, and 7% compared to control samples). Moreover, calpastatin also reduced inclusions in an animal model of MJD (by 68%) [63].

Although calpain inhibitors such as ALLN (MG-101), calpeptin, and BDA-410 have been proposed for SCA3 therapy, there have been no large case studies or clinical trials. However, another calpain inhibitor, olesoxime, has been tested in a Phase 3 trial for ALS [64] and for Huntington’s disease (HD), another polyQ disorder [65].

#### 2.2.3. Inhibition of Ataxin-3 Fragment Nuclear Entry

A related strategy is to inhibit ataxin-3 fragment entry into the nucleus. Bichelmeier and colleages found that transfection of ataxin-3 constructs including the nuclear localization signal resulted in more severe symptoms, a greater number of inclusions, and earlier death of model animals [66]. The transport protein karyopherin α-3 contributes to the shuttling of truncated and full-length expanded ataxin-3 into the nucleus [67], suggesting a major role in SCA3 pathogenesis. However, overexpression of karyopherin α-3 has not been reported in SCA3/MJD patients. In addition, it is likely that karyopherin α-3 regulates the nuclear transport of many other proteins, so inhibition may not be a feasible treatment strategy.

### 2.3. Decreasing Ataxin-3 Aggregation

#### 2.3.1. Phosphorylation/Dephosphorylation of Ataxin-3

Ataxin-3 has multiple phosphorylation sites (S12, S29, S55, T60, S236, S256, S260, S261, S340, and S352) and specific phosphorylation patterns can decrease or increase aggregation. The phosphorylation sites S12, S29, S55, and T60 are in the Josephin domain responsible for catalytic activity, while S236 is within the first ubiquitin-interacting motif (UIM), S256 and S260/261 are within the second UIM, and S340/S352 is in the third UIM [68]. Phosphorylation at S12 decreases aggregation and reduces protein deubiquitination [69]. In contrast, phosphorylation at S29, 340, or 352 by casein kinase 2 (CK2) or glycogen synthase kinase 3 (GSK3) increases protein accumulation in the nucleus, while nuclear inclusions are reduced by inhibition of CK2 or GSK3 [70,71]. Furthermore, CK2 inhibition by DMAT or TBB and GSK3 inhibition using SB216763 decreases ataxin-3 aggregation [35].

The phosphorylation of ataxin-3 at S12 has been detected in MJD patient fibroblasts and healthy controls by Western blotting. Although the MJD patients showed slightly higher phosphorylation levels, the sample sizes were small (*n* = 3 MJD patients and *n* = 1 control) [69], and there is currently insufficient information to speculate on the feasibility of this strategy or the optimal target phosphorylation site(s). Nonetheless, further examination of drug effects on ataxin-3 phosphorylation status and aggregation potential is warranted.

#### 2.3.2. SUMOylation Process of Ataxin-3

The SUMOylation process has been investigated in mutant ataxin-3, but the association with degradation is uncertain. Zhou and colleagues found that the small ubiquitin-like modifier-1 (SUMO-1) stablized mutant ataxin-3 through K166 binding and thereby increased neurotoxicity [72], while Hwang and Lee reported that SUMO-1 binding promotes degradation of ataxin-3 with polyglutamine expansion through enhanced autophagy [73]. Further, SUMOylation at K356 was found to reduce protein aggregation through endoplasmic reticulum (ER)-associated protein degradation [74]. Further experiments are required to clarify the effects of SUMOylation at specific sites on ataxin-3 aggregation, degradation, and clearance.

#### 2.3.3. Autophagy

Targeted autophagic degradation of mutant ataxin-3 has also been proposed as a potential treatment strategy for SCA3/MJD [75,76]. In principle, this therapeutic autophagy could be induced through mammalian target of rapamycin (mTOR)-dependent or -independent pathways [35,76].

Expression of beclin-1, an initiator of mTOR-dependent autophagy, was lower in patient fibroblasts [28,29]. In addition, the LC3II/LC3I ratio was lower and p62 expression higher in patients [28], indicating arrest of autophagic flux in the phagophore without progression to the autophagosome. This may arise from beclin-1 degradation caused by expanded mutant ataxin-3 polyQ, whereas normal ataxin-3 promotes autophagy by preventing proteosome degradation of beclin-1 [77,78].

Rapamycin [30], cordycepin [31], and beclin-1 overexpression can enhance autophagy [29]. Cordycepin activates autophagy by enhancing AMPK activity [31], and this strategy has been applied in a clinical trial for treatment of refractory TdT-positive leukemia (NCT00709215). Rapamycin is a well-known inducer of mTOR-dependent autophagy approved by the FDA and sold under multiple brand names (sirolimus, everolimus, and temsirolimus) to treat several diseases. Rapamycin has not yet been tested in clinical trials. However, the chemical chaperone cabaletta (trehalose), an inducer of mTOR-independent autophogy, has been the subject of one clinical trial for SCA3 (NCT02147886), but did not reduce the SARA score.

Reduced expression of the protein deacetylase Sirt1 has been demonstrated in both SCA3 animal models and patients [32], and the nicotinamide adenine dinucleotide-dependent deacetylase resveratrol can regulate autophagy through increased Sirt1 expression [32]. Caloric restriction or resveratrol rescued SIRT1 expression, induced autophagy, reduced oxidative stress, and improved motor coordination in an animal model [32]. Although resveratrol has not been tested on SCA3 patients, it has been applied for the treatment of Friedreich ataxia, an autosomal recessive disease caused by mutation in the frataxin gene. Resveratrol administration was found to improve neurologic, audiologic, and speech functions, and to reduce oxidative stress markers, but did not alter frataxin levels [79]. Clinical trials of resveratrol for Friedreich ataxia are ongoing (NCT03933163).

#### 2.3.4. Proteosome System

Another strategy to reduce mutant ataxin-3 aggregation is by targeted upregulation of the ubiquitin proteasome pathway (UPS). Proteosomes have been found in nuclear inclusions and shown to suppress polyglutamine aggregation [80]. Drugs such as rho kinase inhibitor (H1152), catalpol, puerarin, and daidzein (the later are active constituents of the medicinal herbs *Rehmannia glutinosa* and *Pueraria lobata*, respectively) have been proposed as potential inducers of ataxin-3 clearance via the UPS [35,81]. Although puerarin has not been applied to neurodegenerative disease, its was shown to improve neurological function and blood perfusion in the ischemic zone of patients with acute cerebral infarction or ischemic stroke [82,83]

However, this strategy is complicated by ataxin-3 function as a deubiquitinating enzyme, and it is unclear whether ataxin-3 or mutant ataxin-3 is a more potent modulator of proteosomal activity. Overexpression of the deubiquitinating enzyme ubiquitin-specific protease 14 (USP14) in HD patients was shown to decrease polyQ protein aggregation [84]. In contrast, USP14 overexpression inhibited the degradation of ubiquitin-mutant ataxin-3 conjugates in vitro, while a small molecule USP14 inhibitor reversed this effect [85], suggesting the possible utility for enhancement of ataxin-3 clearance through proteosomal degradation.

In summary, activation of the proteosome system and autophagy could facilitate the degradation of aggregated ataxin-3. However, effects may be complicated by the deubiquitination activity of ataxin-3 and so this requires further investigation.

#### 2.3.5. Chaperones

Overexpression of protein chaperones, such as heat shock proteins to refold mutant ataxin-3, can reduce aggregation and inclusion formation. Overexpression of HSP40, HSP104, ubiquitin ligase C-terminus of HSP70-interacting protein, DNAJC8, and DNAJB6 have been applied in various disease models characterized by protein inclusions. Furthermore, drugs such as the 17-AAG and 17-DMAG as HSP90 inhibitors, or paeoniflorin (PF), the PF derivative NC001-8, fluorodeoxyuridine, indole, and the indol derivative NC001-8 as heat shock factor (HSF-1), and the HSP70 activators have shown efficacy for decreasing ataxin-3 aggregation [35]

HSP40 recognizes unfolded proteins (substrates) and then combines with HSP70 to form a complex for protein refolding. Zijlstra and colleagues evaluated the expression of the chaperones HSP70 (also termed HSPA1A), HSPA8 (or HSC70), DNAJB (or HSP40), and HSPB1 (or HSP27) in fibroblasts from SCA3 patients and found that both HSP70 and HSP40 expression levels were reduced in early-onset patients [33], strongly suggesting that pathologic ataxin-3 aggregation results in part from a chaperone deficiency. Thus, HSP70 inducers such as PF, the PF derivative NC001-8, and fluorodeoxyuridine could be effective SCA3 treatments. PF is particularly promising as it is a natural Chinese herbal derivative and safety has been confirmed by clinical trials for rheumatoid arthritis [86,87].

An alternative strategy is protecting chaperones from degradation. Aggregation of ATX3Q82 was dramatically reduced in disease model cells co-transfected with DNAJB1 [33]. Evert and co-workers found that miR-370 and miR-543 can target DNAJB1 and are highly expressed in SCA3 patient-derived induced pluripotent stem cell lines [34]. Therefore, protecting DNAJB1 by inhibiting miR-370 and miR-543 may also reduce inclusion formation in SCA3 patients.

### 2.4. Reducing Inflammation and Oxidative Stress

#### 2.4.1. Inhibition of Inflammation

Significant upregulation of the inflammatory markers TNFSF14, FCGR3B, and SELPLG has been detected in MJD patients. Curiously, upregulated expression levels of NFSF14, FCGR3B, CLC, and SLA were found specifically in patients with shorter disease duration (≤9 years), while expression levels were actually reduced in patients with a longer disease course (>10 years) [36]. Elevated expression of eotaxin and increased recruitment of eosinophils to inflammatory sites have also been detected in asymptomatic patients compared to symptomatic patients [88]. This may be attributed to higher expression of neuron-specific enolase (NSE), a peripheral marker of neuronal disruption, in MJD patients [41,42].

Budesonide, an approved drug for maintenance treatment of asthma, improved locomotion and reduced protein aggregation in a *Caenorhabiditis elegans* model of MJD pathogenesis, although it demonstrated relatively low efficiency compared to serotonergic system drugs [16]. Nonetheless, it is still possible that anti-inflammatory drugs will be effective as adjunct therapies. Moreover, the expression of inflammatory factors appears to decrease with disease duration, so anti-inflammatory drugs may be particularly beneficial during the early stages of the disease.

The non-steroidal anti-inflammatory drug ibuprofen [37] has also been examined in SCA3/MJD models and shown to improve neuropathology and motor coordination concomitant with reductions in the neuroinflammatory markers interleukin (IL)-1β, tumor necrosis factor (TNF)-α, and phosphorylated IKB-α. Ibuprofen has been examined in clinical trials for AD and found to reduce delta rhythm progression in mild cases [89]. Moreover, ibuprofen reduced expression levels of the neutrophil markers CD11b and prostaglandin E2 in skin fibroblasts from familial and sporadic AD patients [90].

An alternative anti-inflammatory strategy is to inject neural stem cells [43] as this treatment reduced expression of the proinflammatory markers IL-1β and TNF-α and mitigated Purkinje cell loss.

#### 2.4.2. Mitigation of Oxidative Stress

The ROS-sensitive dye DCFH-DA has revealed more severe oxidative stress in cells from symptomatic SCA3 patients compared to presymptomatic patients or healthy controls (which did not differ). Thus, oxidative stress appears to be directly associated with SCA3 symptom expression. Furthermore, symptomatic patients demonstrated lower endogenous antioxidant capacity as evidenced by reduced expression of superoxide dismutase (SOD) and glutathione peroxidase. More importantly, glutathione peroxidase expression was negatively correlated with the Neurological Examination Score for Spinocerebellar Ataxias [38]. Enhancing cellular antioxidant capacity may therefore slow progression or relieve symptoms. Indeed, overexpression of carbonic anhydrase 8 reduced ROS accumulation and abnormal calcium release in a MJD mouse model [40]. In addition, induced expression of the antioxidant enzyme glutathione S-transferase type 4 by *Brassica napus* (rapeseed) pomace treatment restored motor function in mutant ataxin-3 animals [39]. While rapeseed pomace has not yet been examined in clinical trials, it is likely to show a good safety profile and is easily available as a waste product of rapeseed oil production [91].

### 2.5. Rescue of Cellular Dysfunction

#### 2.5.1. Growth and Neurotrophic Factors

Neuroprotective molecules such as hepatocyte growth factor, brain-derived neurotrophic factor (BDNF), fibroblast growth factor, nerve growth factor [20], and IGF-1 [9] have been applied for SCA3 animal model treatment and assessed in human trials [35,54].

Total IGF-1 did not differ between MJD and control serum samples; however, free circulating IGF-1 (IGF-1:IGFBP-3 molar ratio) was high in SCA3/MJD patients. In addition, reduced insulin expression was observed in SCA3/MJD patients. A higher insulin sensitivity (HOMA2-%S) and a lower resistance index (HOMA2-IR) were also demonstrated in SCA3 patients compared to a control group. Moreover, patients with earlier disease onset exhibited higher HOMA2-%S and lower HOMA2-IR than later-onset patients, supporting a lack of insulin and deficient downstream signaling in SCA3 pathogenesis [44]. However, the low insulin-related symptoms of MJD patients differ from those of type-1 diabetes patients, who exhibit lower IGF-1 [92]. Moreover, β-cell function is well preserved in MJD patients. Nonetheless, upregulation of the insulin receptor in a fly model of SCA3 replenished the cellular pool of CREB binding protein, improved cellular histone acetylation status, and increased autophagy-mediated clearance of polyQ inclusions [45]. This response also differs from diabetes, in which insulin signaling is believed to reduce autophagy [93]

In SCA3 patients, IGF-1 reduced the SARA score after 8 months of treatment but unfortunately worsened the SARA score after 20 months of treatment during a 2-year trial [9]. The rationale for testing IGF-1 treatment is that patients show elevated insulin-like growth factor-binding protein 1 [44], which may inhibit IGF-1-induced cellular responses [94]. The improvement at 8 months may be explained by the activation of the mTOR pathway, ensuing activation of cell proliferation [95]. On the other hand, the deterioration after 20 months of IGF-1 treatment may be caused by (i) the development of IGF-1 resistance, as has been reported in HD patients [92], and/or (ii) decreased autophagy and cell viability under long-term IGF-1 exposure [96].

Neuropeptide Y, which is expressed mainly in the hippocampus, has been proposed as a potential SCA3 treatment based on underexpression in MJD patients and mouse models. Overexpression of neuropeptide Y by injection of an AAV vector improved motor coordination, preserved cerebellar volume and granular layer thickness, increased expression of the neuroprotective factor BDNF, and reduced ataxin-3 aggregation in a SCA3 mouse model [46].

#### 2.5.2. Metabolism

Low glucose metabolism and utilization have been observed in asymptomatic MJD gene carriers [97,98]. In addition, expression levels of the downstream glucose metabolites l-proline and l-tryptophan were lower in symptomatic SCA3 patients compared to asymptomatic patients and healthy controls. Moreover, free fatty acid (FFA) 16:1 (palmitoleic acid) and FFA 18:3 (linolenic acid) expression levels were higher in symptomatic SCA3 patients than healthy controls or presymptomatic SCA3 patients [47]. Collectively, these metabolite deficiencies suggest impaired glucose utilization in SCA3/MJD.

n-Butylidenephthalide (n-BP), a natural compound derived from Angelica sinensis (“female ginseng”), has a variety of disease-fighting properties [48,99,100]. It also has the capacity to reduce activity of the tryptophan-metabolizing enzyme tryptophan 2, 3-dioxygenase (TDO2) [48]. Plasma tryptophan is elevated in TDO2-deficient (Tdo−/−) mice [101], so n-BP may be useful for maintaining tryptophan quantity and 5-HT signaling in SCA3/MJD patients.

#### 2.5.3. Enzymes

Cholesterol 24S-hydroxylase (CYP46A1), the rate-limiting enzyme in cholesterol degradation, was reduced in cerebellar extracts from SCA3 patients and in SCA3 mice, whereas AAV-mediated overexpression of CYP46A1 protected cerebellar PCs, reduced ataxin-3 protein aggregation, and improved autophagy [49]. In addition, CYP46A1 enhanced the proteosomal and autophasomal degradation of mutant huntingtin aggregates [102]. Furthermore, CYP46A1 can be activated by Efavirenz, an anti-retroviral medication reported to improve learning and memory by reducing excess cholesterol in synapses during long-term potentiation [103,104].

#### 2.5.4. Transcription Regulation

Normal ataxin-3 promotes expression of the antioxidant enzyme SOD2 by interacting with the transcription factor FOXO4 [105]. In addition, ataxin-3 interacts with human Rad23, a protein that may translocate proteolytic substrates to the proteasome [106]. In contrast, mutant ataxin-3 reduces SOD2 expression and delivery of proteolytic substrates to the proteasome. In addition, the polyQ stretches of mutant ataxin-3 can bind TAFII130 and strongly suppress CREB-dependent transcriptional activation [107]. Therefore, transcriptional activators as well as promoters of proteosomal degradation may serve as SCA3 treatments. For instance, HDAC inhibitors such as sodium butyrate, VPA, and suberoylanilide hydroxamic acid rescue hypoacetylated histone H3 and H4 levels, thereby enhancing transcription. In addition, nicotinamide and nicotinamide adenine dinucleotide, which inhibit class III HDACs, have also been proposed as SCA3 treatments.

However, wild-type ataxin-3 can also suppress transcription by binding to HDAC3. Also, increased HDAC activity was found in mice overexpressing normal ataxin-3, whereas reduced HDAC activity was found in mutant ataxin-3 mice [108]. Therefore, there is currently insufficient understanding of transcriptional regulation via ataxin-3/HDAC pathways to speculate on the potential effects of HDAC inhibitors on SCA3-related processes.

### 2.6. Neuronal Homeostasis

#### 2.6.1. Glutamate Receptor Signaling

Retinoid-related orphan receptor α (RORα), a type 1 metabotropic glutamate receptor signaling molecule [109], sustains PC dendritic complexity and mono-innervation by climbing fibers [110]. Decreased RORα expression was detected in the nuclei of SCA3 mouse PCs [109,111], and single injection of the RORα/γ agonist SR1078 rescued the behavioral, morphological, and functional deficits in these SCA3 model mice [111].

#### 2.6.2. Ion Channel Homeostasis

SCA3 model cells also exhibit potassium channel dysfunction as evidenced by reduced (depolarized) resting membrane potential [112]. Based on such findings, the calcium-activated potassium (SK) channel agonists SKA-31 and riluzole have been proposed as treatments for SAC3/MJD [53]. Indeed, SKA-31 improved motor function in SCA3 model mice. Riluzole also suppresses glutamate release and thus reduces AMPA receptor-mediated depolarization and the ensuing voltage-dependent calcium influx [53]. A clinical trial from 2015 reported that riluzole reduced the SARA score in 50% of patients treated [13]. However, riluzole in the drinking water was reported to have no beneficial effect and possibly even an adverse effect on transgenic SCA3 model mice [52]. These results suggest the importance of drug formulation, delivery route, and pharmacokinetics. Perhaps oral administration and subsequent exposure to the low pH of the digestive tract alters the pharmaceutical properties.

Mutant ataxin-3 triggers the release of calcium from the ER through activation of the inositol 1,4,5-trisphosphate receptor. The G-protein-coupled purinergic receptor P2RY13, which also induces intracellular calcium release [113], is upregulated in the sera of MJD patients. Thus, suppressing ER calcium release and influx may slow the progression of neurodegeneration in MJD. The widely used muscle relaxant dantrolene is known to suppress calcium signaling through inhibition of ryanodine receptor-mediated intracellular release, and was shown to improve motor performance and prevent neuronal cell loss in the pontine nuclei and substantia nigra of SCA3 model mice [50]. While these preclinical studies were started in 2008, there are yet no clinical trials on dantrolene for treatment of SCA3.

#### 2.6.3. Adenosinergic System

Expression levels of adenosine and the A2A receptor have been examined in HD, but very little is known about the contributions of adenosinergic signaling in SCA3. Expression of the A2A receptor is reduced in the putamen of HD patients, and disrupted BDNF production or transport in HD was rescued by stimulation of A2A receptors using an exogenous agonist or endogenous adenosine. Moreover, activated adenosine A2A receptors can promote the expression of prosurvival genes and the UPS through the AMPK signaling pathway [114]. Oral administration of T1–11, an extract of the Chinese medicinal herb *Gastordia elata* that acts as an adenosin A2A receptor agonist, or the synthetic T2-ll analog JMF1907 improved motor coordination, decreased the expression of ataxin-3, and enhanced the chymotrypsin-like activity of proteasomes in SCA3 transgenic mice [115]. In addition, caffeine has been demonstrated to reduce synaptotoxicity and gliosis caused by mutant ataxin-3 via the adenosine A2A receptor [116].

#### 2.6.4. Serotonergic System

Teixeira-Castro and colleagues screened FDA-approved modulators of neurotransmission, including adrenergic, serotonergic, cholinergic, dopaminergic, and histaminergic agents, as well as anti-inflammatory drugs, cardiovascular drugs, hormones/hormone substituents, analgesics, and anti-infective agents for effects on protein aggregation and locomotion in a *C. elegans* model of SCA3. They found that serotonergic agents significantly improved locomotion deficits and reduced insoluble aggregated ataxin-3 protein. Among serotonergic molecules, the synaptic 5-HT transport inhibitor citalopram demonstrated greatest efficacy [16]. Dihydroergotamine, which promotes serotonin release by inhibiting presynaptic 5-HT autoreceptors, had similar effects on locomotion as citalopram [16]. In addition, the 5-HT2C receptor agonist vabicaserin also improved locomotion, although not as effectively as dihydroergotamine [16].

As described in the section on metabolism, the 5-HT precursor tryptophan is reduced in serum samples from MJD patients, suggesting that 5-HT synthesis and release may be compromised [47]. However, neither 5-HT level nor 5-HT turnover (5-HIAA/5-HT ratio) in the cerebellum, medulla oblongata, and substantia nigra differed between symptomatic MJD mice (24 weeks old) and wild-types [16]. Compared with wild-type mice, the expression of tryptophan hydroxylase (TPH) was only lower in the presymptomatic stage (4 weeks of age) and continued to be underexpressed to 12 weeks of age in HD model mice [117]. These results may explain the reason why there is no difference between symptomatic MJD mice (24 weeks old) and wild types. Moreover, decreased 5-HT1A receptor binding affinity was observed in 12-week-old R6/2 huntingtin (HD model) mice [117], which may explain why inhibition of the 5-HT transporter by citalopram is more effective than 5-HT receptor activation using vabicaserin in SCA3 animal models [16].

An alternative strategy for 5-HT modulation is cell therapy. Intracranial human olfactory ensheathing cell transplantation prevented PC loss and enhanced motor coordination through increased expression of TPH2 [118].

## 3. Discussion

We have summarized the development and testing of drugs for treating SCA3/MJD, including neurotransmitter release modulators, growth factors, HDAC inhibitors, autophagy enhancers, and stem cells. Moreover, we have reviewed biological markers and associated pathogenic processes identified in preclinical studies that may provide clues to novel treatments for SCA3/MJD.

The most studied drugs for SCA3 treatment are those that suppress glutamate-induced neuronal calcium increases, including riruzole, riruzole derivatives, and TRH agonists. However, the evidence for glutamate-induced neurotoxicity (excitotoxicity) in SCA3 progression is largely derived from in vitro studies of cellular models and in vivo studies of SCA3 model animals [119,120,121,122], and few known biomarkers provide a rationale for such studies with the exception of those showing elevated intracellular calcium [113] due to altered expression of the adenosinergic receptor P2RY13 [36]. More targeted treatments for calcium dysregulation in SCA3 may emerge from future comparative genomics and proteomics studies.

Agonists of the 5-HT1A receptor were the first drugs used for SCA3 treatment and have shown promising results in some smaller-scale trials; however, there have been few recent studies on their efficacy. Blockers of 5-HT reuptake and antagonists of presynaptic autoreceptors are also promising agents to enhance serotonergic transmission in the cerebellum. The 5-HT precursor tryptophan was reduced in SCA3 patients [47] and the biosynthetic enzyme TPH was downregulated in young animal models of HD, like SCA3, a disorder characterized by polyQ protein inclusions [117]. Reduced 5HT1A receptor ligand-binding was also observed in HD model mice [117]. Hence, serotonergic transmission may be disrupted by abnormalities in multiple steps along the biosynthesis–release–postsynaptic receptor response pathway. Therefore, the relative efficacies of 5-HT receptor agonists, uptake inhibitors, autoreceptor antagonists, and agents that upregulate 5-HT biosynthesis will depend on the mechanisms underlying 5-HT transmission deficits.

Inclusions of misfolded mutant ataxin-3 is the pathological hallmark of SCA3; therefore, many preclinical studies have focused on enhancing misfolded protein clearance/degradation. Autophagy is a major protein degradation and recycling pathway under stress, and, as such, has attracted intense research interest as a potential mechanism for reducing polyQ protein aggregation. Moreover, autophagy markers are consistently underexpressed in SCA3 patients [28,29], further supporting enhanced autophagy as a potential strategy for SCA3 treatment. A related strategy is the induction of molecular chaperones that assist in protein refolding or degradation. Like biomarkers of autophagy, chaperones such as HSC70 and DNAJB (HSP40) are underexpressed in SCA3/MJD patients. The chemical chaperone mTOR-independent autophagy inducer trehalose is a particularly promising candidate and has been examined in Phase 2 clinical trials.

There is also evidence for deficient gene translation or protein transcription in SCA3. In principle, a general deficit in protein production could be reversed by HDAC inhibitors, and indeed one such drug (VPA) has been tested on SCA3 patients. However, this treatment is nonspecific and is further complicated by the endogenous HDAC function of ataxin-3. Much additional preclinical research may be required before this strategy is feasible for SCA3 treatment.

Lower glucose metabolism due to disrupted insulin signaling [9] also appears to participate in SCA3/MJD pathogenesis [97,98]. One major consequence of this deficit is reduced tryptophan availability [47], which in turn may contribute to the aforementioned 5-HT signaling deficits [117]. Insulin-like growth factor treatment appeared to improve SCA3 patient condition after 8 months [9]; however, the gains disappeared within 20 months. Alternatively, increased insulin receptor activity enhanced autophagy-mediated removal of polyQ inclusions [45] but did not alter β-cell function in SCA3/MJD patients [44]. Collectively, these findings suggest that enhancing glucose utilization may improve SCA3 by increasing both insulin and serotonin production. On the other hand, FFA 16:1 (palmitoleic acid) and FFA 18:3 (linolenic acid) were increased in SCA3/MJD patients [47]. This high palmitoleic acid improved insulin sensitivity among type 2 diabetes mellitus patients [123] and prevented reduced insulin sensitivity in non-diabetic individuals [124], which adversely support the finding from patients with high levels of HOMA2-%S [44]. In addition, symptomatic SCA3 patients also exhibit increased expression of monounsaturated fatty acids and polyunsaturated fatty acids [47], and previous studies have shown that both act as antidepressants [125]. We speculate that these elevations may be compensatory and reduce disease-associated depression.

Relief of neuroinflammation is another potential SCA3 treatment strategy. However, inflammatory factors were detected only in SCA3 patients with a disease course shorter than 9 years (possibly stemming from ROS generation by aggregated proteins or removal mechanisms). However, many inflammatory factors were below baseline in patients more than 10 years after disease onset. We speculate that this eventual loss of inflammatory activity results from progressively reduced glucose metabolism and energy production, as evidenced by the low levels of tryptophan and proline in symptomatic patients 8.2 years after disease onset [47].

Finally, miRNAs, siRNA, shRNA may be employed for targeted modulation of disease-related mRNAs and proteins. The development of these therapeutic strategies requires further study of optimal doses and treatment periods.

In conclusion, disease biomarkers may reveal novel therapeutic strategies or combinations for general or stage-specific treatment of SCA3/MJD. Autophagy induction via small molecule drugs or miRs could be applied as first-line treatment to remove the pathological proteins, followed by drugs that enhance glucose metabolism to power self-repair mechanisms and supply required substrates, such as tryptophan for 5-HT biosynthesis. Finally, suppressors of neuroinflammation, oxidative stress, and excitotoxicity may be applied to slow neurodegeneration and alleviate motor symptoms.

## Figures and Tables

**Table 1:** Clinical trials for treating spinocerebellar ataxia type 3/Machado–Joseph disease (SCA3/MJD).

Indication	Drug Name	Mechanisms	Status	Outcome	NCT no.	Year
Cerebellar ataxia	Buspirone	5-HT1A serotonin agonist	Case-study/completed	Improved gait ataxia	-	1994 [6]
SCA3	Tandospirone	5-HT1A serotonin agonist	Case-study/completed	Leg pain, insomnia, anorexia, and depression remarkably alleviated	-	1994 [7]
SCA3	Tandospirone	5-HT1A serotonin agonist	Double-blind study/completed	1. ARS reduction in 7/10 patients. 2. SDS reduction in 3/6 patients. 3. Insomnia and leg pain alleviation in 5/7 patients.	-	2001 [8]
SCA3 and SCA7	Insulin-like growth factor-1 (IGF-1)	Neuromodulatory functions	Open label/completed	SARA improved after 8 months and worsened after 20 months.	-	2007 [9]
SCA3	Varenicline	agonist at α4β2 neuronal nicotinic acetylcholine receptors	Phase 2/completed	1. Side effect of nausea. 2. Improved axial symptoms and rapid alternating movements.	NCT00992771	9 Oct 2009 [10]
SCA3	Sodium phenylbutyrate	HDAC inhibitors	Withdrawn ^#^	-	NCT01096095	30 Mar 2010
SCA3	VPA	HDAC inhibitors	Phase 1/completed	SARA score (−2.05) greater in the VPA group than in the placebo (−0.75) groups	ChiCTR-TRC10000754	6 Jan 2010 [11]
SCA3	Lithium carbonate	Interfere with ion transport processes	Phase 2/Phase 3/completed	No effect on progression (NESSCA)	NCT01096082	30 Mar 2010 [12]
Cerebellar Ataxia	Riluzole	Glutamate release inhibitor	Phase 2/Phase 3/completed	1. 50% patient with decrease SARA score. 2. No severe adverse events were recorded	NCT01104649	15 Apr 2010 [13]
SCA3	NGF	Neuroprotection	Open label/Completed	Total SARA score decreased significantly	-	Nov 2011
Cerebellar ataxia	Allogeneic adult Ad-MSC	Neuroprotection	Phase 1/Phase 2/completed	1. No adverse events 2. Increased brain glucose metabolism	NCT01649687	25 Jul 2012 [14]
SCA3	Cabaletta (trehalose)	Chemical chaperone	Phase 2, completed	Stable on the SARA scale.	NCT02147886	28 May 2014
SCA1, 2, 3, and 6	Dalfampridine	Potassium channel blocker	Completed	No difference in change of T25FW and SARA score	NCT01811706	12 Jan 2015
Cerebellar Ataxia	Stemchymal^®^	Neuroprotection	Unknown, Phase 2		NCT02540655	4 Sep 2015
SCA1, 2, 3, and 6	hUC-MSC	-	Phase 2, unknown	-	NCT03378414	19 Dec 2017
SCA1, 2, 3, 6, 7, 8, and 10	Troriluzole	Glutamate release inhibitor	Phase 3	-	NCT03701399	10 Oct 2018
SCA1, 2, 3, 6, and MSA-C	BHV-4157 (pro-drug of riluzole)	Glutamate release inhibitor	Phase 3, active, not recruiting	-	NCT03408080	23 Jan 2018
Ataxia, Cerebellar	Nilotinib (Bcr-Abl TKI)	Autophagy enhancer	Phase 2, active, not recruiting	-	NCT03932669	1 May 2019
Spinocerebellar Degeneration	C-Trelin OD Tab (analogue of TRH)	Inhibition of activation of glutamate	Recruiting, Phase 4	-	NCT04107740	27 Sep 2019

^#^: Regulatory authorities did not allow the entrance of the study drug in the country. Abbreviation: Machado–Joseph disease (MJD), ataxia rating scale (ARS), self-rating depression scale (SDS), Neurological Examination Score for the Assessment of Spinocerebellar Ataxia (NESSCA), nerve growth factor (NGF), Timed 25 Feet Walking Test (T25FW), adipose-derived mesenchymal stem cells (Ad-MSC), human umbilical cord mesenchymal stem cells (hUC-MSC), thyrotropin releasing hormone(TRH), tyrosine kinase inhibitor (TKI), histone deacetylation (HDAC), valproic acid (VPA).

**Table 2:** Combination of the information from biological markers of SCA3/MJD patients and the current therapeutic strategies against those expressed biological markers.

Mechanism	Biomarkers	Function	Expressions Level in Subject	Treatment	Therapy Results
RNAi-mediated knockdown of ataxin-3					
Target to ataxin-3	MiR-25	Bind to ATXN3 3′-UTR	Underexpressed in SCA3 patients [21]	MiR-25 mimics	Suppressed 3′UTR of ATXN3 mRNA [22]
Target to ataxin-3	Mir-9	Bind to ATXN3 3′-UTR	Underexpressed in SCA3 patients (CSF-derived exosome and neurons) [23,24].	miRNA overexpression	Suppressed 3′UTR of ATXN3 mRNA [23]
Target to ataxin-3	Mir-181a	Bind to ATXN3 3′-UTR	Underexpressed in SCA3 patients (CSF-derived exosome and neurons) [23,24].	miRNA overexpression	Suppressed 3′UTR of ATXN3 [23]
Target to ataxin-3	Mir-494	Bind to ATXN3 3′-UTR	Underexpressed in SCA3 patients (neurons) [23].	miRNA overexpression	Suppressed 3′UTR of ATXN3 [23]
Reduced cleavage protein formation					
Calpain inhibitor	Calpastatin	Calpain inhibitor	Underexpressed in SCA3 patients [25].	ALLN (MG-101) or calpeptin	Reduced full-length and small fragment ataxin-3 via Calpeptin [26]. Reduced small fragment ataxin-3 via ALLN [27].
Decreasing ataxin-3 aggregation					
Autophagy	Beclin-1	Autophagy initiator	Underexpressed in symptomatic SCA3 patients [28,29]	Beclin-1 overexpression	mTOR-dependent pathways activation [28,29]
Autophagy	Ratio of LC3II/LC3I	Autophagosome	Underexpressed in SCA3 patient’s fibroblasts [28]	Rapamycin or cordycepin	mTOR-dependent pathways activation [28,29,30,31]
Autophagy	P62	Deliver ubiquitinated proteins	Higher in SCA3 patient’s fibroblasts [28]	Rapamycin or cordycepin	mTOR-dependent pathways activation [28,29,30,31]
Autophagy	Sirtuin-1	NAD ^+^-dependent deacetylase	Underexpressed in SCA3 patient’s fibroblasts [32]	Caloric restriction or resveratrol	Rescuing SIRT1 levels, motor incoordination, imbalance [32]
Chaperon	DNAJB1	Protein refolding machine	Significantly Underexpressed in SCA3 with early-onset patients [33]. Underexpressed in SCA3 patient-derived iPSC lines [34]	DNAJB1 overexpression [33]	Largely reduced ATX3Q82 aggregation in HEK cell [33]
Chaperon	HSPA1A	Protein refolding machine	Underexpressed in SCA3 patient’s fibroblast [33]	Paeoniflorin (PF), PF derivative NC001-8, or Fluorodeoxyuridine	Enhancing the expression of HSF-1 and HSP70 chaperones [35]
Chaperon	HSPA8	Protein refolding machine	Underexpressed in SCA3 patient’s fibroblast [33]	Paeoniflorin (PF), PF derivative NC001-8, or Fluorodeoxyuridine	Enhancing the expression of HSF-1 and HSP70 chaperones [35]
Reducing inflammation and oxidative stress					
Inflammatory factors	TNFSF14	Neurodegenerative	Higher in SCA3 patients with duration ≤9 years [36]	Ibuprofen [37]	Reduced Il1b, TNFa mRNA and IKB-α protein phosphorylation levels [37]
Oxidative Stress	SOD	Antioxidant enzyme activities	Underexpressed in symptomatic SCA3 [38]	RSP [39], or CA8 overexpression [40]	Induction of GST-4iva RSP [39]. Rescued abnormal Ca^2+^ release via CA8overexpression [40].
Oxidative Stress	GSH-Px	Antioxidant enzyme activities	Underexpressed in symptomatic SCA3 [38]	RSP [39], or CA8 overexpression [40]	Induction of GST-4iva RSP [39]. Rescued abnormal Ca^2+^ release via CA8overexpression [40].
Neural degeneration	NSE	Peripheral marker of neuronal disruption	Higher in SCA3 [41,42]	Neural stem cells injection [43]	Decreased pro-inflammatory mediators IL1B and TNFA [43]
Rescue of cellular dysfunction.					
Growth factors	Insulin	Growth factors	Underexpressed in SCA3 [44]	IGF-1	Significantly decreased in SARA scores [9]
Growth factors	IGF-1/IGFBP-3	Free IGF-1	Higher in SCA3 [44]	Insulin receptor Upregulation [45]	Increased autophagy-mediated to rescue phenotype [45]
Neurotrophic	Neuropeptide Y	Neuroprotective molecule	Underexpressed in SCA3 [46]	NPY overexpression [46]	Increased BDNF levels [46]
Metabolism	Tryptophan	Amino acid metabolism	Underexpressed in SCA3 [47]	n-BP [48]	Decreased TDO2 expression [48]
Enzyme	CYP46A1	brain cholesterol turnover Activation	Underexpressed in SCA3 [49]	CYP46A1 overexpression [49]	Decreased aggregation ataxin-3 protein and increased Purkinje cell number [49]
Ion-channel homostatasis	P2RY13	Increase of intracellular calcium	Higher in SCA3 patients [36]	Dantrolene [50], SKA-31 [51], or riluzole [52]	Inhibited calcium release from ER via dantrolene [50]. Activated Kv3.1 channels via SKA-31 [51]. Prevented calcium influx increase in the cells via riluzole [53].

Abbreviations: rapeseed pomace (RSP), carbonic anhydrase 8 (CA8), superoxide dismutase (SOD), and glutathione peroxidase (GSH-Px), neuron-specific enolase (NSE), n-butylidenephthalide (n-BP), brain-derived neurotrophic factor (BDNF), purinergic receptor P2Y, G-protein coupled 13 (P2RY13), DnaJ homolog subfamily B member 1 (DNAJB1), induced pluripotent stem cell (iPSC), cholesterol 24-hydroxylase (CYP46A1) glutathione S-transferase (GST-4), heat shock factor (HSF-1).
